# Outcomes of infantile leukemia in a Saudi multicenter cohort: patterns of treatment response, treatment failure, relapse, and survival

**DOI:** 10.3389/fonc.2026.1871807

**Published:** 2026-07-20

**Authors:** Abdulmajeed Abdulrahman, Ibrahim Ghemlas, Hawazen Al-Saedi, Ali Al-Ahmari, Saadiya Khan, Khawar Siddiqui, Wasil Jastaniah, Sara Ramiz

**Affiliations:** Department of Paediatrics Haematology/Oncology, King Faisal Specialist Hospital and Research Centre, Riyadh, Saudi Arabia

**Keywords:** acute lymphoblastic leukemia, acute myeloid leukemia, infantile leukemia, relapse, survival outcomes, treatment failure, treatment response

## Abstract

**Background:**

Infantile leukemia is a rare and aggressive form of pediatric leukemia associated with high rates of treatment failure, relapse, and mortality. Despite advances in leukemia therapy, evidence from Saudi Arabia remains limited, particularly regarding treatment response and survival outcomes among infants with acute lymphoblastic leukemia (ALL) and acute myeloid leukemia (AML).

**Aim:**

This study aimed to describe treatment response, treatment failure, relapse, overall survival, and event-free survival among infants with acute leukemia treated at two tertiary referral centers in Saudi Arabia, and to explore outcome differences according to leukemia subtype.

**Methods:**

A retrospective multicenter observational cohort study was conducted at King Faisal Specialist Hospital and Research Centre in Riyadh and Jeddah. The study included 41 patients diagnosed with infantile leukemia between January 2012 and December 2021. Data were extracted from medical records using a structured data collection form. Demographic, clinical, treatment-response, relapse, treatment-failure, and survival outcomes were summarized using descriptive statistics. Comparisons between ALL and AML were performed using Fisher’s exact test where appropriate because of small cell counts. Overall survival and event-free survival were estimated using Kaplan–Meier analysis and compared using the log-rank test.

**Results:**

Of the 41 patients, 24 (58.5%) had ALL and 17 (41.5%) had AML. At last follow-up, 22 patients (53.7%) were alive and 19 (46.3%) had died. Among patients evaluable after first-line therapy, 20 of 26 (76.9%) achieved first complete remission. Disease reactivation or relapse occurred in 11 patients (26.8%), death in remission in 6 (14.6%), and treatment failure or refractory disease in 6 (14.6%). Median overall survival was not reached for the total cohort, while median event-free survival was 23.27 months. No statistically significant differences were observed between ALL and AML in overall survival, log-rank p = .930, or event-free survival, log-rank p = .778.

**Conclusion:**

Infantile leukemia was associated with considerable adverse outcomes in this Saudi multicenter cohort, including relapse, death in remission, treatment failure, and mortality. Leukemia subtype alone was not significantly associated with survival or adverse outcome patterns. These findings should be interpreted as descriptive and hypothesis-generating because of the small sample size and limited statistical power.

## Introduction

Leukemia remains the most common malignant disease in childhood and continues to represent a major cause of cancer-related morbidity and mortality among children and adolescents worldwide (1). Although advances in diagnosis, risk stratification, chemotherapy protocols, supportive care, hematopoietic stem cell transplantation, and targeted therapies have improved outcomes, survival remains variable across age groups, leukemia subtypes, biological profiles, and health-care settings (1, 2). This variability highlights the need for continued evaluation of clinical characteristics, treatment response, relapse patterns, treatment failure, and survival outcomes in pediatric leukemia populations (3). Such evaluation is particularly important for rare subgroups in which evidence remains limited and treatment outcomes are difficult to generalize from larger pediatric leukemia cohorts.

Infantile leukemia, commonly defined as leukemia diagnosed during the first year of life, is a rare but biologically aggressive form of pediatric leukemia (4). It differs from leukemia in older children in terms of clinical presentation, molecular characteristics, treatment tolerance, and prognosis (4, 5). Infants with leukemia are particularly vulnerable because of developmental immaturity, increased susceptibility to treatment-related toxicity, and frequent association with high-risk cytogenetic and molecular abnormalities, including KMT2A rearrangements in a substantial proportion of infant acute lymphoblastic leukemia cases (5). Neonatal leukemia represents an even rarer subgroup and is often associated with distinctive hematologic and extramedullary manifestations that require rapid diagnosis and specialized management (6). These unique features support the need to evaluate infantile leukemia separately from leukemia occurring in older pediatric age groups (4).

Treatment failure and relapse remain major challenges in pediatric acute leukemia because they are strongly associated with inferior survival outcomes (3). In infant leukemia, younger age and induction failure have been reported as important predictors of poor outcome (7). Studies among children with acute lymphoblastic leukemia have also shown that treatment failure is associated with clinical and laboratory factors reflecting disease aggressiveness and early response to therapy (8, 9). In pediatric B-cell acute lymphoblastic leukemia with KMT2A rearrangements, poor treatment response has been linked to poor outcomes, emphasizing the prognostic importance of biological and early-response markers (1, 17–19). Therefore, describing treatment response, relapse, treatment failure, and survival patterns in infantile leukemia is essential for generating clinically relevant evidence and identifying areas for future risk-stratified research.

Risk stratification has become central to modern leukemia management because it allows treatment intensity to be adapted according to the expected risk of treatment failure, relapse, and mortality (11). In infant acute lymphoblastic leukemia, risk-adapted therapeutic approaches have been evaluated to improve survival while minimizing unnecessary treatment-related toxicity (11). However, relapse continues to be one of the strongest determinants of long-term survival in acute lymphoblastic leukemia (3). Outcomes after relapse are influenced by disease characteristics, prior treatment exposure, timing of relapse, and response to salvage therapy (3). Emerging immunotherapeutic approaches, including CD19 CAR T-cell therapy, have created new treatment opportunities for relapsed or refractory disease, although preinfusion factors may influence relapse phenotype and treatment outcomes (12). These developments highlight the importance of local outcome data to support future studies that incorporate clinical, cytogenetic, molecular, and treatment-related variables.

Despite the growing international literature on pediatric and infantile leukemia, evidence from Middle Eastern populations, including Saudi Arabia, remains limited (1–3). Survival outcomes, relapse patterns, treatment failure, and treatment response may vary across populations because of differences in genetic background, referral pathways, treatment protocols, supportive care resources, and access to specialized oncology services (2). Regional studies are therefore needed to determine whether internationally reported outcome patterns are applicable to Saudi patients with infantile leukemia (7, 11). Saudi-specific multicenter evidence is also important for characterizing local clinical and biological patterns that may inform future risk stratification and treatment decision-making (4, 5, 14–16). Accordingly, this study aimed to describe treatment response, treatment failure, relapse, overall survival, and event-free survival among infants with acute leukemia treated at two tertiary referral centers in Saudi Arabia, and to explore outcome differences according to leukemia subtype.

## Methodology

### Research design

This study employed a retrospective, multicenter, observational cohort design to describe treatment response, treatment failure, relapse, overall survival, and event-free survival among patients diagnosed with infantile leukemia in Saudi Arabia. The retrospective design was appropriate because the study relied on previously documented clinical, diagnostic, treatment, and follow-up data extracted from medical records. The study was observational and non-interventional, as no changes were made to patient management or treatment protocols. A multicenter approach was used to include patients treated at two tertiary referral hospitals in Saudi Arabia, allowing broader evaluation of clinical characteristics and outcomes in this rare patient population.

### Study setting

The study was conducted at two tertiary referral hospitals in Saudi Arabia: King Faisal Specialist Hospital and Research Centre in Riyadh and King Faisal Specialist Hospital and Research Centre in Jeddah. Both centers provide specialized pediatric hematology and oncology services and manage complex cases of acute leukemia, including acute lymphoblastic leukemia and acute myeloid leukemia. The multicenter setting was selected to increase the number of eligible cases and provide a broader representation of infantile leukemia outcomes among patients treated in specialized oncology centers in Saudi Arabia.

### Research population

The research population consisted of patients diagnosed with infantile leukemia, including acute lymphoblastic leukemia (ALL) and acute myeloid leukemia (AML), who received treatment at the participating tertiary hospitals in Saudi Arabia. Infantile leukemia was defined as leukemia diagnosed within the first year of life. The accessible population included all eligible patients treated during the study period from January 2012 to December 2021 and for whom relevant diagnostic, clinical, treatment, outcome, and follow-up data were available in the medical records.

### Research sample

The research sample included all eligible patients diagnosed with infantile leukemia, including ALL and AML, who were treated at King Faisal Specialist Hospital and Research Centre in Riyadh or Jeddah between January 2012 and December 2021. A total of 41 patients were included in the final analysis, comprising 24 patients with ALL and 17 patients with AML. Because infantile leukemia is rare, all eligible cases during the study period were included rather than selecting a sample from the eligible population.

### Inclusion and exclusion criteria

Patients were included if they were diagnosed with infantile leukemia, specifically ALL or AML, within the first year of life and received treatment at one of the participating centers between January 2012 and December 2021. Eligible patients were required to have sufficient medical-record data to confirm diagnosis, leukemia subtype, survival status, and follow-up outcome. Patients with Down syndrome or other congenital anomalies were not excluded, as the study aimed to describe infantile leukemia as a whole cohort. These conditions were recorded as clinical variables and were planned for descriptive reporting and exploratory subgroup analysis where numbers permitted. Patients were excluded only if they had unavailable essential diagnostic or survival data, or if they were diagnosed with secondary leukemia, such as leukemia occurring after previous chemotherapy exposure.

### Data collection procedure

Data were collected retrospectively from patients’ medical records at the participating centers. Eligible cases were identified from hospital records, pediatric hematology/oncology databases, and electronic medical records of patients diagnosed with ALL or AML during the study period from January 2012 to December 2021. After identifying eligible patients, clinical charts and electronic medical records were reviewed to extract the required data using a structured case report form.

The collected data included demographic characteristics, leukemia subtype, clinical and pathological features, cytogenetic and molecular findings where available, treatment protocol, treatment response, relapse status, treatment failure or refractory disease, stem cell transplantation status, survival status, and follow-up duration. Data from King Faisal Specialist Hospital and Research Centre in Jeddah were obtained through the assigned site co-investigator according to institutional data confidentiality and privacy policies. After data extraction, the dataset was reviewed, cleaned, and checked for missing values, inconsistencies, duplicate entries, and abnormal values before statistical analysis.

### Candidate variables

Based on clinical relevance and availability in retrospective records, the candidate variables considered for descriptive reporting and exploratory subgroup comparison included leukemia subtype, age at diagnosis, sex, baseline leukocyte count, central nervous system involvement, cytogenetic and molecular abnormalities where available, treatment protocol, first-line treatment response, stem cell transplantation status, relapse, treatment failure or refractory disease, survival status, event-free survival status, and follow-up duration.

Because of the small sample size and limited number of outcome events, the study was not powered to identify independent prognostic risk factors. Therefore, candidate variables were summarized descriptively, and inferential analysis was limited to selected exploratory comparisons, mainly according to leukemia subtype.

### Evaluability after first-line therapy

Patients were considered evaluable after first-line therapy if post-treatment response assessment was documented in the medical record, including complete remission, refractory disease, progressive disease, or early death after first-line therapy. Patients without sufficient documentation of post-treatment response were considered non-evaluable for this specific outcome but were retained in the overall cohort and included in survival analyses when survival status and follow-up data were available.

Among the 41 included patients, 26 patients were evaluable after first-line therapy. The remaining 15 patients were not included in the first-line treatment-response analysis because formal response assessment was not available or could not be reliably determined from the retrospective records. Patients without sufficient documentation of formal post-treatment response assessment were classified as non-evaluable for first-line treatment response. Because the specific reason for non-evaluability could not be reliably categorized for all cases from the retrospective records, these patients were excluded only from the first-line treatment-response analysis and were retained in survival analyses when survival status and follow-up data were available.

### Outcome measures

The primary outcome measures were treatment response, treatment failure, relapse, overall survival, and event-free survival among patients diagnosed with infantile leukemia. Treatment failure was defined as failure to achieve complete remission after first-line therapy or the presence of refractory or progressive disease. Relapse was defined as disease reactivation after achieving remission, including bone marrow, central nervous system, or other documented relapse sites. Death in remission was defined as death occurring after documented remission without evidence of active relapse or refractory disease.

Overall survival was measured from the date of diagnosis to the date of death from any cause or last follow-up for patients who were alive. Event-free survival was measured from the date of diagnosis to the first documented event, including treatment failure, relapse, death in remission, or last follow-up for patients without an event. Secondary variables included leukemia subtype, treatment response, remission status, cytogenetic and molecular abnormalities where available, treatment protocol, stem cell transplantation status, and follow-up duration.

### Missing data handling

Missing data were assessed for each extracted variable before analysis. Variables with incomplete documentation were summarized using available-case denominators, and the number of available observations was reported where appropriate. No statistical imputation was performed because of the retrospective design, small sample size, and expected non-random nature of missing clinical documentation. Patients with missing data for a specific variable were excluded only from analyses involving that variable and were retained in all other applicable analyses. Variables with substantial missingness were not included in inferential analysis and were interpreted descriptively.

### Ethical considerations

This study was conducted after obtaining the required institutional and ethical approvals from the participating centers. The study was reviewed and approved by the Research Advisory Council at King Faisal Specialist Hospital and Research Centre, approval number RAC 2241338. As the study was retrospective and based on review of existing medical records, no direct contact with patients or families was required, and no intervention was performed. A waiver of informed consent was obtained because the study involved previously documented clinical data. Patient confidentiality was maintained throughout the study by using coded data and limiting access to the research team only. No identifiable patient information was included in the analysis, reports, or manuscript. All data were collected, stored, and analyzed in accordance with the institutional policies of King Faisal Specialist Hospital and Research Centre regarding data privacy, confidentiality, and security.

### Data analysis

Data were entered, cleaned, and analyzed using IBM SPSS Statistics. Descriptive statistics were used to summarize demographic, clinical, pathological, cytogenetic, treatment-related, and outcome characteristics. Categorical variables were presented as frequencies and percentages. Continuous variables were summarized using means and standard deviations for normally distributed data or medians and ranges/interquartile ranges for non-normally distributed data.

Comparisons between ALL and AML were performed as exploratory analyses. Associations between categorical variables and outcome categories were assessed using Fisher’s exact test because of the small sample size and expected small cell counts. The chi-square test was used only when its assumptions were met. Comparisons of continuous variables between groups were performed using the independent-samples t-test for normally distributed variables or non-parametric tests for skewed variables, as appropriate.

Overall survival and event-free survival were estimated using Kaplan–Meier survival analysis, and survival distributions were compared between ALL and AML using the log-rank test. Median survival was reported where reached. When median survival was not reached, this was stated, and fixed-time survival estimates such as 3-year and 5-year survival rates were reported where available. Although mean survival estimates were generated by Kaplan–Meier analysis, interpretation focused primarily on median survival and survival probabilities because these are more appropriate for censored survival data.

Given the rarity of infantile leukemia, the small cohort size, and the limited number of outcome events, multivariable Cox proportional hazards regression and logistic regression were not performed. Therefore, the analysis was considered descriptive and exploratory rather than confirmatory for independent prognostic risk factors. A p-value of less than 0.05 was considered statistically significant.

## Results

A total of 41 patients diagnosed with infantile leukemia were included in the analysis. Of these, 24 patients (58.5%) were diagnosed with acute lymphoblastic leukemia (ALL), and 17 patients (41.5%) were diagnosed with acute myeloid leukemia (AML). At the last follow-up, 22 patients (53.7%) were alive, whereas 19 patients (46.3%) had died. Regarding event-free survival status, 18 patients (43.9%) were alive with no event, 11 patients (26.8%) had disease reactivation or relapse, 6 patients (14.6%) died in remission, and 6 patients (14.6%) had treatment failure or refractory disease (**Table 1**).

**Table 1:** Baseline diagnosis and outcome characteristics of the cohort.

Variable	Category	Frequency, n	Percentage, %
Diagnosis group	Acute lymphoblastic leukemia (ALL)	24	58.5
	Acute myeloid leukemia (AML)	17	41.5
Survival status at last follow-up	Alive	22	53.7
	Died	19	46.3
Event-free survival status	Alive, no event	18	43.9
	Disease reactivation/relapse	11	26.8
	Death in remission	6	14.6
	Treatment failure/refractory disease	6	14.6

Completeness of data varied across the extracted variables. Leukemia subtype, survival status at last follow-up, and event-free survival status were available for all 41 patients. First-line treatment response was evaluable in 26 patients (63.4%), while 15 patients (36.6%) had missing or insufficient documentation for formal first-line response assessment. Cytogenetic/molecular data, detailed treatment protocol information, and stem cell transplantation-related variables were incompletely documented in the retrospective records and could not be reliably quantified across the full cohort. Therefore, these variables were summarized descriptively where available and were not included in inferential analysis. Available records showed that 7 patients with AML underwent stem cell transplantation, but SCT status, indications, and post-transplantation outcomes were not consistently documented for the total cohort. No statistical imputation was performed, and available-case denominators were used for variables with missing data (**Table 2**).

**Table 2:** Summary of missing data for key study variables.

Variable	Available data, n/N (%)	Missing data, n/N (%)	Handling in analysis
Leukemia subtype	41/41 (100.0)	0/41 (0.0)	Included in descriptive and exploratory analyses
Survival status at last follow-up	41/41 (100.0)	0/41 (0.0)	Included in overall survival analysis
Event-free survival status	41/41 (100.0)	0/41 (0.0)	Included in event-free survival analysis
First-line treatment response	26/41 (63.4)	15/41 (36.6)	Available-case analysis
Cytogenetic/molecular data	Incompletely documented and not reliably quantifiable across the full cohort	Incompletely documented and not reliably quantifiable across the full cohort	Summarized descriptively where available; excluded from inferential analysis
Detailed treatment protocol	Incompletely documented and not reliably quantifiable across the full cohort	Incompletely documented and not reliably quantifiable across the full cohort	Summarized descriptively where available; excluded from inferential analysis
Stem cell transplantation status	Partially documented; 7 AML patients were recorded as having undergone SCT	Incompletely documented for the total cohort	Reported descriptively using available records

Available-case analysis was used for variables with incomplete documentation. No statistical imputation was performed because of the retrospective design, small sample size, and non-random nature of missing clinical documentation. SCT, stem cell transplantation.

### First-line treatment response and relapse outcomes

Of the 41 included patients, 26 patients (63.4%) were evaluable after first-line therapy, including 17 patients with ALL (70.8%) and 9 patients with AML (52.9%). The remaining 15 patients (36.6%) were not evaluable for first-line treatment response because formal post-treatment response assessment was not sufficiently documented in the retrospective medical records. The available records suggested that non-evaluability was mainly related to incomplete documentation of formal post-treatment response assessment, absence of clearly recorded post-induction bone marrow response, transfer of care or treatment continuation outside the participating center, or other clinical circumstances that prevented reliable classification of first-line response. Because the specific reason could not be reliably categorized for every non-evaluable case, these patients were excluded only from the first-line treatment-response analysis and were retained in survival and outcome analyses when survival status and follow-up data were available.

Among evaluable patients, 20 of 26 patients (76.9%) achieved first complete remission (CR-1). The proportion achieving CR-1 was 14 of 17 patients (82.4%) among those with ALL and 6 of 9 patients (66.7%) among those with AML. First-line therapy failure or progressive disease occurred in 5 of 26 evaluable patients (19.2%), including 2 patients with ALL (11.8%) and 3 patients with AML (33.3%). Early death after first-line therapy was reported in 1 of 26 evaluable patients (3.8%), who belonged to the ALL group.

Disease reactivation or relapse occurred in 11 patients (26.8%) overall, including 7 patients with ALL (29.2%) and 4 patients with AML (23.5%). Death in remission was reported in 6 patients (14.6%), comprising 4 patients with ALL (16.7%) and 2 patients with AML (11.8%). Treatment failure or refractory disease was documented in 6 patients (14.6%), including 3 patients with ALL (12.5%) and 3 patients with AML (17.6%). At last follow-up, 18 patients (43.9%) were alive with no event, including 10 patients with ALL (41.7%) and 8 patients with AML (47.1%). Stem cell transplantation data were incompletely documented for the total cohort; however, 7 patients with AML (41.2%) were reported to have undergone stem cell transplantation (**Table 3**).

**Table 3:** Treatment response and relapse outcomes according to leukemia subtype.

Outcome variable	Total, n = 41	ALL, n = 24	AML, n = 17
Evaluable after first-line therapy	26 (63.4)	17 (70.8)	9 (52.9)
Achieved CR-1 among evaluable cases	20/26 (76.9)	14/17 (82.4)	6/9 (66.7)
Failed first-line therapy/progressive disease among evaluable cases	5/26 (19.2)	2/17 (11.8)	3/9 (33.3)
Early death after first-line therapy	1/26 (3.8)	1/17 (5.9)	0/9 (0.0)
Disease reactivation/relapse	11 (26.8)	7 (29.2)	4 (23.5)
Death in remission	6 (14.6)	4 (16.7)	2 (11.8)
Treatment failure/refractory disease	6 (14.6)	3 (12.5)	3 (17.6)
Alive with no event	18 (43.9)	10 (41.7)	8 (47.1)
Stem cell transplantation	7/available n (%)	Not fully reported	7 (41.2)

Values are presented as n (%) unless otherwise indicated. ALL = acute lymphoblastic leukemia; AML = acute myeloid leukemia; CR-1 = first complete remission. Available-case denominators are shown where not all patients had evaluable data. Stem cell transplantation data were incompletely documented for the total cohort.

### Exploratory comparison of adverse outcomes by leukemia subtype

Fisher’s exact test was used to explore whether adverse outcomes differed between patients with ALL and AML. Death occurred in 19 patients (46.3%), including 11 patients with ALL (45.8%) and 8 patients with AML (47.1%). There was no statistically significant association between leukemia subtype and death, OR = 0.952, p = 1.000.

Any event-free survival event occurred in 23 patients (56.1%), including 14 patients with ALL (58.3%) and 9 patients with AML (52.9%). The association between leukemia subtype and any event-free survival event was not statistically significant, OR = 1.244, p = .760. Similarly, leukemia subtype was not significantly associated with disease reactivation or relapse, death in remission, treatment failure/refractory disease, or being alive with no event (**Table 4**). The overall distribution of event-free survival outcomes, categorized as alive with no event, disease reactivation/relapse, death in remission, and treatment failure/refractory disease, did not differ significantly between patients with ALL and AML, Pearson χ²(3, N = 41) = 0.527, p = .913.

**Table 4:** Exploratory comparison of adverse outcomes according to leukemia subtype.

Outcome variable	Total, n = 41	ALL, n = 24	AML, n = 17	Odds ratio	P-value
Death/overall survival event	19 (46.3)	11 (45.8)	8 (47.1)	0.952	1.000
Any EFS event	23 (56.1)	14 (58.3)	9 (52.9)	1.244	0.760
Disease reactivation/relapse	11 (26.8)	7 (29.2)	4 (23.5)	1.338	0.736
Death in remission	6 (14.6)	4 (16.7)	2 (11.8)	1.500	1.000
Treatment failure/refractory disease	6 (14.6)	3 (12.5)	3 (17.6)	0.667	0.679
Alive with no event	18 (43.9)	10 (41.7)	8 (47.1)	0.804	0.760

Values are presented as n (%). ALL, acute lymphoblastic leukemia; AML, acute myeloid leukemia; EFS, event-free survival. Fisher’s exact test was used for binary comparisons because of small cell counts. The overall EFS outcome distribution compared four categories: alive with no event, disease reactivation/relapse, death in remission, and treatment failure/refractory disease. These analyses were exploratory and were not intended to identify independent prognostic risk factors.

### Overall survival and event-free survival

Kaplan–Meier analysis was used to estimate overall survival and event-free survival according to leukemia subtype. For overall survival, 19 deaths occurred in the total cohort (including 6 deaths in remission, comprising 4 patients with ALL and 2 patients with AML; the remaining deaths occurred outside documented remission and should be further classified, where data are available, as relapse-related, refractory/progressive disease–related, or unclear), while 22 patients (53.7%) were censored.

The median overall survival was not reached for the total cohort or for patients with ALL, while the median overall survival among patients with AML was 33.53 months. The mean overall survival estimate was 82.58 months for the total cohort, 79.04 months for patients with ALL, and 81.43 months for patients with AML. However, because survival data were censored, interpretation focused primarily on median survival and survival distribution. There was no statistically significant difference in overall survival between patients with ALL and AML using the log-rank test, χ²(1, N = 41) = 0.008, p = .930.

For event-free survival, 23 events occurred in the total cohort, while 18 patients (43.9%) were censored. Among patients with ALL, 14 EFS events occurred and 10 patients (41.7%) were censored. Among patients with AML, 9 EFS events occurred and 8 patients (47.1%) were censored. The median event-free survival was 23.27 months for the total cohort, 12.33 months for patients with ALL, and 30.30 months for patients with AML. Although patients with AML had a numerically longer median EFS than patients with ALL, this difference was not statistically significant, log-rank χ²(1, N = 41) = 0.080, p = .778 (**Table 5**).

**Table 5:** Kaplan–Meier survival outcomes according to leukemia subtype.

Survival outcome	Comparison group	Total N	Events, n	Censored, n (%)	Mean survival, months	95% CI	Median survival, months	Log-rank χ²	P-value
Overall survival	ALL	24	11	13 (54.2)	79.04	51.95–106.14	Not reached	0.008	0.930
	AML	17	8	9 (52.9)	81.43	47.12–115.74	33.53		
	Overall	41	19	22 (53.7)	82.58	60.51–104.65	Not reached		
Event-free survival	ALL	24	14	10 (41.7)	62.52	35.63–89.41	12.33	0.080	0.778
	AML	17	9	8 (47.1)	71.04	36.09–105.99	30.30		
	Overall	41	23	18 (43.9)	68.06	45.92–90.20	23.27		

ALL, acute lymphoblastic leukemia; AML, acute myeloid leukemia; CI, confidence interval. Overall survival events were defined as death from any cause. Event-free survival events included disease reactivation/relapse, death in remission, and treatment failure/refractory disease. Survival distributions between ALL and AML were compared using the log-rank test. Mean survival estimates are reported as Kaplan–Meier outputs but should be interpreted cautiously because of censored survival data. Median overall survival was not reached for the total cohort or ALL group because the largest survival times were censored.

### Kaplan–Meier curves

**Figure 1:**
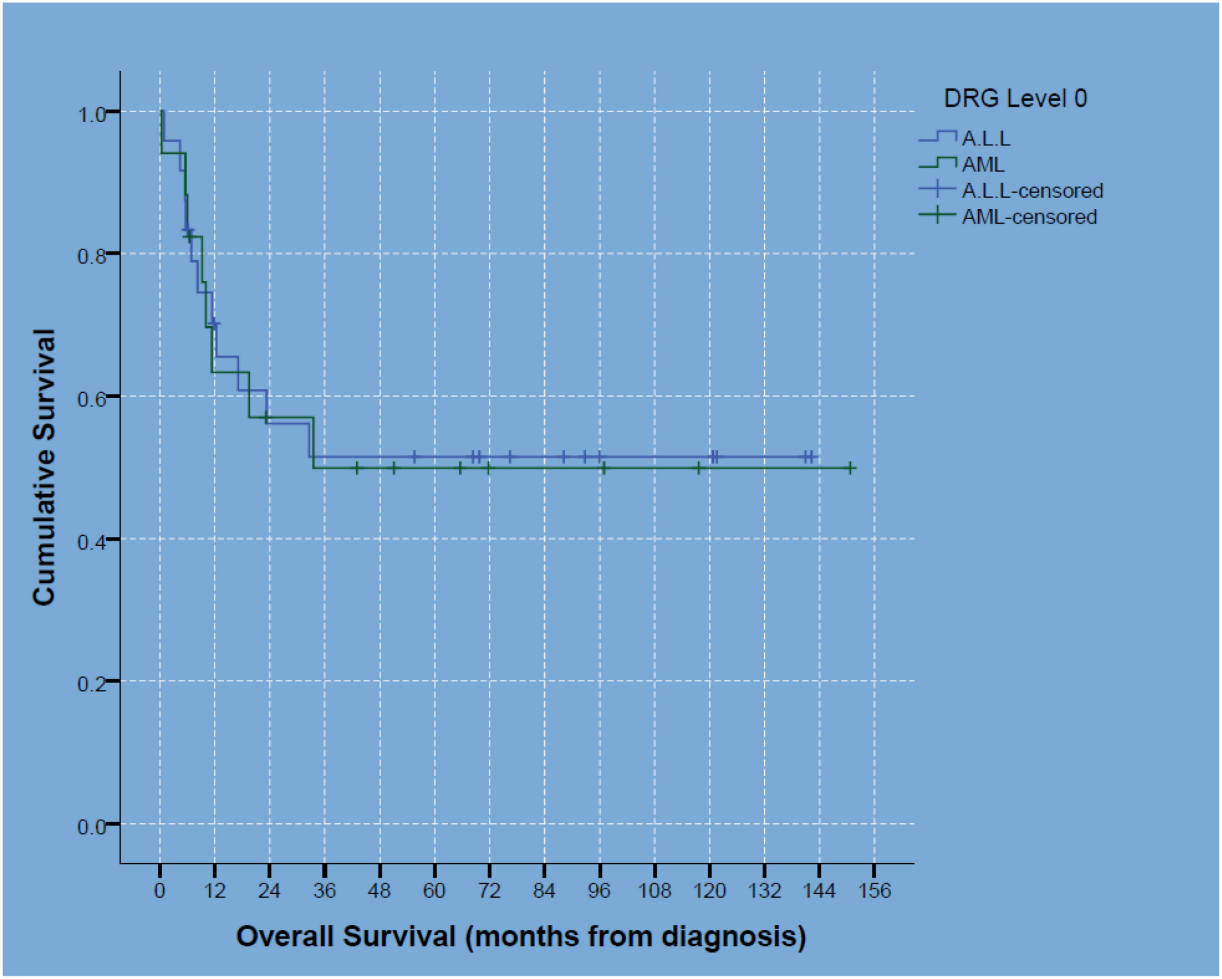
Kaplan–Meier curve for overall survival according to leukemia subtype.

**Figure 1** presents the Kaplan–Meier curve for overall survival according to leukemia subtype. The survival curves for patients with ALL and AML showed a generally similar pattern across the follow-up period, with no statistically significant difference between groups, log-rank χ²(1, N = 41) = 0.008, p = .930.

**Figure 2:**
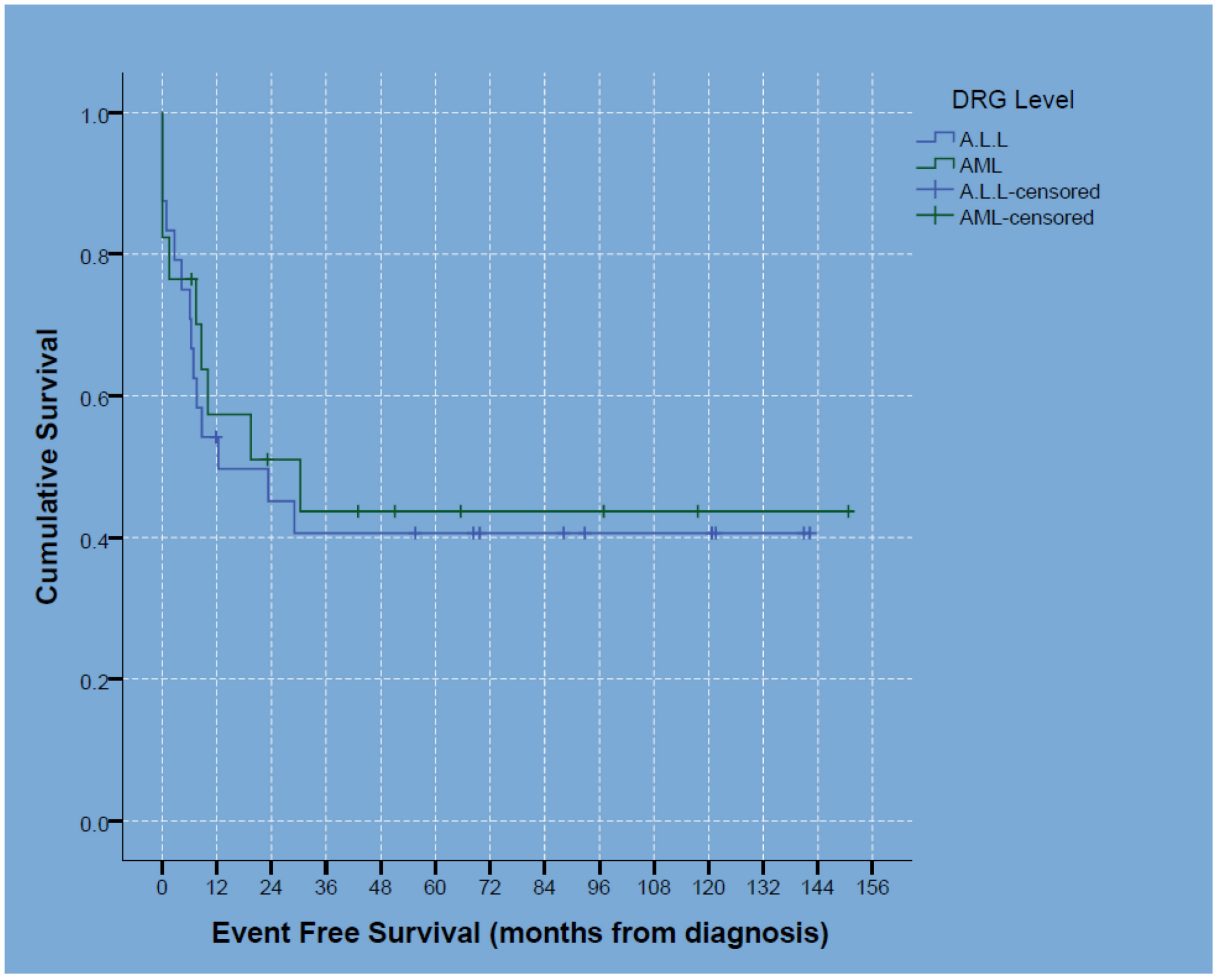
Kaplan–Meier curve for event-free survival according to leukemia subtype.

**Figure 2** presents the Kaplan–Meier curve for event-free survival according to leukemia subtype. The EFS curves for both ALL and AML showed an early decline during the initial follow-up period, indicating that many events occurred relatively early after diagnosis. Although patients with AML showed a numerically longer median EFS than patients with ALL, the difference was not statistically significant, log-rank χ²(1, N = 41) = 0.080, p = .778.

## Discussion

The present study described treatment response, treatment failure, relapse, overall survival, and event-free survival among infants with acute leukemia treated at two tertiary referral centers in Saudi Arabia. The cohort included 41 patients, with ALL representing the larger proportion of cases compared with AML. This distribution is consistent with the general pattern of pediatric leukemia, in which ALL is more frequent than AML, although infantile leukemia remains biologically and clinically distinct from leukemia occurring in older children (4, 5). The findings showed that infantile leukemia was associated with a substantial burden of adverse outcomes, including relapse, death in remission, treatment failure or refractory disease, and mortality. These results support the need for continued evaluation of infantile leukemia outcomes in Saudi Arabia, particularly because local multicenter data remain limited.

The rate of adverse events observed in this cohort reflects the aggressive nature of infantile leukemia. More than half of the patients experienced an event during follow-up, including disease reactivation or relapse, death in remission, or treatment failure/refractory disease. This finding is clinically important because infantile leukemia is frequently associated with high-risk biological features, limited treatment tolerance, and increased vulnerability to treatment-related complications (5, 13). Although the current study was not powered to identify independent prognostic predictors, the observed outcome burden highlights the need for risk-adapted management strategies and systematic national data collection for this rare population.

Regarding early treatment response, most evaluable patients achieved first complete remission after first-line therapy. The CR-1 rate was numerically higher among patients with ALL than among those with AML. This finding suggests that initial treatment response was achieved in a considerable proportion of evaluable cases; however, a clinically important subgroup still experienced first-line therapy failure, progressive disease, or early death. Previous studies have emphasized that induction failure and poor early response are strongly associated with inferior outcomes in infant and pediatric leukemia (7, 8). The higher proportion of first-line therapy failure or progressive disease among AML cases in this cohort may reflect the aggressive biology and treatment complexity of infant AML, although this interpretation should be cautious because of the small number of evaluable AML patients.

Relapse and treatment failure remained central adverse outcomes in this cohort. Disease reactivation or relapse occurred in more than one-quarter of patients, while treatment failure or refractory disease was reported in a smaller but clinically meaningful proportion. These findings are consistent with previous evidence showing that relapse after initial therapy is one of the strongest determinants of long-term survival in acute leukemia (3). In infant ALL, high-risk genetic lesions, including KMT2A rearrangements, have been associated with treatment resistance and relapse risk (10, 13). However, because cytogenetic and molecular data were not completely available for all patients in the current cohort, these biological factors could not be robustly evaluated as predictors of relapse or treatment failure. Future Saudi studies should therefore include standardized reporting of cytogenetic, molecular, minimal residual disease, and treatment-response variables.

Kaplan–Meier analysis showed no statistically significant difference between ALL and AML in overall survival or event-free survival. Although AML showed a numerically longer median event-free survival than ALL, this difference did not reach statistical significance. This finding should not be interpreted as evidence that ALL and AML have equivalent biological risk in infants. Rather, it likely reflects the small sample size, limited number of events within each subgroup, and heterogeneity in clinical and treatment-related characteristics. Survival outcomes in childhood leukemia are influenced by multiple factors, including age at diagnosis, leukemia subtype, cytogenetic and molecular abnormalities, initial leukocyte count, treatment protocol, treatment response, minimal residual disease, relapse timing, stem cell transplantation, and supportive care resources (2, 3, 11). Therefore, leukemia subtype alone may be insufficient to explain outcome variation in infantile leukemia.

The exploratory comparison of adverse outcomes by leukemia subtype also showed no statistically significant association between subtype and mortality, relapse, treatment failure/refractory disease, death in remission, any event-free survival event, or being alive with no event. These findings should be interpreted as descriptive and hypothesis-generating rather than confirmatory. The study did not include multivariable Cox regression or logistic regression because of the small sample size and limited number of outcome events. Therefore, confounding by clinical, biological, and treatment-related variables could not be controlled. This limitation is important because infantile leukemia prognosis is likely shaped by a combination of biological and treatment-response factors rather than diagnosis group alone.

The findings also highlight the importance of improving the completeness and standardization of infantile leukemia data collection in Saudi Arabia. Review of the available records showed incomplete documentation for some clinically important variables, including detailed cytogenetic and molecular findings, treatment protocols, and stem cell transplantation-related information. These variables are important because they may influence relapse, treatment failure, and survival. For example, KMT2A rearrangement status, central nervous system involvement, baseline leukocyte count, minimal residual disease, treatment protocol, and transplantation status are all clinically relevant candidate predictors that should be included in future prospective or registry-based studies. Establishing a national infant leukemia registry would allow more reliable evaluation of outcome patterns and independent prognostic factors in Saudi patients.

This study contributes to the limited regional evidence by describing treatment response, relapse, treatment failure, and survival outcomes among infants with leukemia treated at specialized Saudi referral centers. The multicenter design strengthens the relevance of the findings, particularly because infantile leukemia is rare and difficult to study in single-center cohorts. However, the findings should be interpreted within the context of a descriptive retrospective design. The study provides useful local outcome data and identifies areas requiring future investigation, but it does not establish independent risk factors for adverse outcomes.

Several limitations should be considered when interpreting the findings. First, the retrospective design depended on the accuracy and completeness of medical records, which limited the availability of some clinical, cytogenetic, molecular, treatment, transplantation, and follow-up variables. Second, the sample size was small because infantile leukemia is rare. This reduced statistical power to detect differences between ALL and AML and prevented robust multivariable analysis. Third, the number of events was limited within each outcome category, particularly treatment failure and death in remission, which restricted the ability to perform Cox proportional hazards regression or logistic regression. Fourth, first-line treatment response was evaluable in only 26 patients, and non-evaluability may have introduced selection bias if patients without response assessment differed clinically from evaluable patients. In addition, the specific reasons for non-evaluability could not be reliably categorized for all non-evaluable cases because of the retrospective nature of documentation.

Fifth, some treatment-related data, including detailed protocols and stem cell transplantation indications and outcomes, were incompletely documented. Finally, although the study was multicenter, it included two specialized tertiary referral centers, which may limit generalizability to all hospitals in Saudi Arabia.

Despite these limitations, the study provides important descriptive evidence on a rare and high-risk pediatric leukemia population in Saudi Arabia. The findings suggest that infantile leukemia is associated with substantial rates of relapse, treatment failure, death in remission, and mortality. They also indicate that leukemia subtype alone did not explain outcome differences in this cohort. Larger national studies with standardized collection of clinical, cytogenetic, molecular, treatment-response, and transplantation-related variables are needed to identify independent predictors of treatment failure, relapse, overall survival, and event-free survival.

## Conclusion

Infantile leukemia was associated with substantial adverse outcomes in this Saudi multicenter cohort, including relapse, death in remission, treatment failure or refractory disease, and mortality. ALL was the most common leukemia subtype, and more than half of the patients experienced an event during follow-up. Although patients with AML showed a numerically longer event-free survival than patients with ALL, no statistically significant differences were observed between the two leukemia subtypes in overall survival, event-free survival, relapse, treatment failure, or mortality.

These findings suggest that leukemia subtype alone may not adequately explain outcome variation among infants with acute leukemia. However, the results should be interpreted as descriptive and exploratory because of the small sample size, limited statistical power, and incomplete availability of some biological and treatment-related variables. Larger multicenter or national studies in Saudi Arabia are needed to integrate clinical, cytogenetic, molecular, treatment-response, and transplantation-related data to identify independent predictors of treatment failure, relapse, and survival, and to support more individualized risk stratification and management strategies.

## Data Availability

The raw data supporting the conclusions of this article will be made available by the authors, without undue reservation.

## References

[1] Cui Y Yan Y . The global burden of childhood and adolescent leukaemia and attributable risk factors: An analysis of the Global Burden of Disease Study 2019. J Global Health. (2024) 14:4045. doi: 10.7189/jogh.14.04045 38426852 PMC10906348

[2] Bordbar M Jam N Karimi M Shahriari M Zareifar S Zekavat OR . The survival of childhood leukemia: An 8-year single-center experience. Cancer Rep. (2023) 6:e1784. doi: 10.1002/cnr2.1784 36700480 PMC10075287

[3] Rheingold SR Bhojwani D Ji L Xu X Devidas M Kairalla JA . Determinants of survival after first relapse of acute lymphoblastic leukemia: A Children’s Oncology Group study. Leukemia. (2024) 38:2382–94. doi: 10.1038/s41375-024-02395-4 39261601 PMC11518984

[4] Ibrahimova A Pommert L Breese EH . Acute leukemia in infants. Curr Oncol Rep. (2021) 23:27. doi: 10.1007/s11912-021-01021-1 33580326

[5] Wertheim G . Infant acute leukemia. Clinics Lab Med. (2021) 41:541–50. doi: 10.1016/j.cll.2021.04.002 34304781

[6] Brown PA . Neonatal leukemia. Clinics Perinatol. (2021) 48:15–33. doi: 10.1016/j.clp.2020.11.002 33583502

[7] Ochoa-Fernández B Galán-Gómez V Guerra-García P Sanromán S Martínez I Bueno D . Younger age and induction failure predict outcomes in infant leukemia: 30 years of experience in a tertiary center. Front Pediatr. (2023) 11:1166176. doi: 10.3389/fped.2023.1166176 37325355 PMC10263122

[8] Gao W Jiang MY Gao L Lu J Xiao PF He HL . The factors related to treatment failure in children with acute lymphoblastic leukemia. Zhongguo Shi Yan Xue Ye Xue Za Zhi. (2021) 29:661–8. doi: 10.19746/j.cnki.issn.1009-2137.2021.03.002 34105454

[9] Meng-Meng YIN Qun HU Ai-Guo LIU Ya-Qin WANG Ai ZHANG . Factors associated with prognosis and treatment failure in children with acute lymphoblastic leukemia. Chin J Contemp Pediatr. (2025) 27:308. doi: 10.22541/au.161658415.52847149/v1 40105076 PMC11928036

[10] Wen J Zhou M Shen Y Long Y Guo Y Song L . Poor treatment responses were related to poor outcomes in pediatric B cell acute lymphoblastic leukemia with KMT2A rearrangements. BMC Cancer. (2022) 22:859. doi: 10.1186/s12885-022-09804-w 35933338 PMC9357304

[11] Tomizawa D Miyamura T Imamura T Watanabe T Moriya Saito A Ogawa A . A risk-stratified therapy for infants with acute lymphoblastic leukemia: A report from the JPLSG MLL-10 trial. Blood. (2020) 136:1813–23. doi: 10.1182/blood.2019004741 32845001

[12] Lamble AJ Myers RM Taraseviciute A John S Yates B Steinberg SM . Preinfusion factors impacting relapse immunophenotype following CD19 CAR T cells. Blood Adv. (2023) 7:575–85. doi: 10.1182/bloodadvances.2022007423 35482927 PMC9979750

[13] Kulczycka M Derlatka K Tasior J Sygacz M Lejman M Zawitkowska J . Infant acute lymphoblastic leukemia—New therapeutic opportunities. Int J Mol Sci. (2024) 25:3721. doi: 10.3390/ijms25073721 38612531 PMC11011884

[14] Benítez L Castro-Barquero S Crispi F Youssef L Crovetto F Fischer U . Maternal lifestyle and prenatal risk factors for childhood leukemia: A review of the existing evidence. Fetal Diagn Ther. (2024) 51:395–410. doi: 10.1159/000539141 38710162

[15] Huang FL Liao EC Li CL Yen CY Yu SJ . Pathogenesis of pediatric B-cell acute lymphoblastic leukemia: Molecular pathways and disease treatments. Oncol Lett. (2020) 20:448–54. doi: 10.3892/ol.2020.11583 32565969 PMC7285861

[16] Kintossou AK Blanco-Lopez J Iguacel I Pisanu S Almeida CCB Steliarova-Foucher E . Early life nutrition factors and risk of acute leukemia in children: Systematic review and meta-analysis. Nutrients. (2023) 15:3775. doi: 10.3390/nu15173775 37686807 PMC10489830

[17] Onyije FM Olsson A Baaken D Erdmann F Stanulla M Wollschlaeger D . Environmental risk factors for childhood acute lymphoblastic leukemia: An umbrella review. Cancers. (2022) 14:382. doi: 10.3390/cancers14020382 35053543 PMC8773598

[18] Schmidt JA Hornhardt S Erdmann F Sánchez-García I Fischer U Schüz J . Risk factors for childhood leukemia: Radiation and beyond. Front Public Health. (2021) 9:805757. doi: 10.3389/fpubh.2021.805757 35004601 PMC8739478

[19] Schraw JM Bailey HD Bonaventure A Mora AM Roman E Mueller BA . Infant feeding practices and childhood acute leukemia: Findings from the Childhood Cancer & Leukemia International Consortium. Int J Cancer. (2022) 151:1013–23. doi: 10.1002/ijc.34062 35532209 PMC12893794

